# Variations on a Theme: Two Structural Motifs Create Species-Specific Pheromone Channels for Multiple Species of South American Cerambycid Beetles

**DOI:** 10.3390/insects11040222

**Published:** 2020-04-02

**Authors:** Weliton D. Silva, Lawrence M. Hanks, Jean Carlos S. Alvarez, Fernando Z. Madalon, José Maurício S. Bento, Jan E. Bello, Jocelyn G. Millar

**Affiliations:** 1 Department of Entomology and Acarology, University of São Paulo, Piracicaba, SP 13418900, Brazil; 2 Department of Entomology, University of Illinois at Urbana-Champaign, Urbana, IL 61801, USA; 3 Academic Department of Agronomy, National University of Tumbes, Tumbes 24001, Peru; 4 Departments of Entomology and Chemistry, University of California, Riverside, CA 92521, USA

**Keywords:** Coleoptera, longhorn beetles, semiochemistry, aggregation-sex pheromone, monitoring

## Abstract

We describe the identification, synthesis, and field-testing of aggregation-sex pheromones, or likely pheromone candidates, of seven species of South American cerambycid beetles in the subfamily Cerambycinae, of the tribes Eburiini and Neoibidionini. Analyses of extracts of volatiles released by adult males revealed that *Eburodacrys dubitata* White produce 11-methyltridecanal, whereas the males of *Eburodacrys assimilis* Gounelle, *Eburodacrys flexuosa* Gounelle, and *Eburodacrys lenkoi* Napp and Martins produce blends of this compound, along with its analog 10-methyldodecanal. In contrast, males of *Compsibidion graphicum* (Thomson) and *Compsibidion sommeri* (Thomson) produce blends of 10-methyldodecanal and its corresponding alcohol 10-methyldodecanol. The results from field bioassays with synthetic compounds showed that each species was specifically attracted to traps containing their reconstructed pheromone blend. However, *E. assimilis* was not trapped, possibly due to inhibition by non-natural enantiomers in the racemic test compounds. During the trials for the *Compsibidion* species, adults of another cerambycid species, *Tetraopidion mucoriferum* (Thomson), were captured in significant numbers in traps baited with 10-methyldodecanol, suggesting that this compound is a pheromone component for this species. This study demonstrates another case of conservation of pheromone structures within South American cerambycid species. It also highlights how blends of closely related structures, differing only in chain length or functional group, make the evolution of species-specific pheromone channels possible.

## 1. Introduction

Our knowledge of the semiochemistry of the large beetle family Cerambycidae has advanced rapidly over the past 15 years, with several dozen pheromone components with quite varied structures being identified from several hundred species worldwide [1]. Work has advanced particularly rapidly for cerambycid species in the subfamilies Cerambycinae, Lamiinae, and Spondylidinae, whose adult males produce relatively large quantities of so-called aggregation-sex pheromones [2], which bring conspecific males and females together on their host plants for mating and reproduction [3]. A remarkable feature of the chemistry of these compounds is that some structures appear to be highly conserved among both closely related (e.g., congeners) and more distantly related species (e.g., species in different subfamilies), in which the beetles may produce similar or even identical pheromone components [1]. For example, many cerambycine species use pheromones consisting of 6, 8, or 10 carbon chains with ketone or alcohol groups at C2 and C3, or alcohols in both positions [1]. Similarly, hydroxyethers such as monochamol (2-[undecyloxy]ethanol), and terpenoid derivatives such as fuscumol ([*E*]-6,10-dimethyl- 5,9-undecadien-2-ol) and fuscumol acetate ([*E*]-6,10-dimethyl-5,9-undecadien-2-yl acetate) are used as pheromone components by a number of lamiine and spondylidine species [1]. Additionally, this parsimony in biosynthesis and the use of similar structural motifs as pheromones often extends beyond continental boundaries, with compounds such as 3-hydroxyhexan-2-one being used as pheromone components by cerambycid species endemic to all continents except Antarctica [3], indicating that this and other shared structures have been conserved within the family for millions of years.

From a practical viewpoint, this parsimony implies that multiple cerambycid species may be attracted to traps baited with single components or blends of related pheromone components [4,5,6,7,8,9,10]. As such, these pheromones are finding increasing use in the delineation of the geographic ranges of native species [11], and in surveillance programs for detecting exotic species [12]. In particular, wood-boring cerambycid beetles are of major importance as potential invaders, because of the ease with which they are transported between continents in wooden products and packing materials by global commerce [9,10,12]. This parsimony also implies that the identification of pheromone components for one cerambycid species may subsequently expedite the identification of pheromones or likely pheromones for related target species [8,13,14].

By contrast, some cerambycid species appear to produce uncommon or even unique pheromone motifs, which may be species-specific or used by a limited number of species. For example, (*Z*)-3-decenyl (*E*)-2-hexenoate is the male-produced aggregation-sex pheromone of the North American species *Rosalia funebris* Motschulsky, and this compound has not attracted any other cerambycid species in field trials in North America, Asia, or Europe [1]. Similarly, 10-methyldodecanal [15] and (*Z*)-7-hexadecene [16] have been identified as aggregation-sex pheromone components of the South American species *Eburodacrys vittata* (Blanchard) and *Susuacanga octoguttata* (Germar), respectively, and these compounds have hitherto not attracted any additional species in field trials in Brazil [16] or the eastern United States (LMH unpublished data).

However, this apparent species-specificity can be misleading, because pheromones are often blends, in which single components of the blend may have little or no activity. Here, we present evidence that functionalized 10-methyldodecanes, along with the homologous functionalized 11-methyltridecanes, are shared pheromone components of at least eight South American cerambycid species in the subfamily Cerambycinae.

## 2. Materials and Methods

The eight cerambycid study species included five *Eburodacrys* species (tribe Eburiini), two *Compsibidion* species (Neoibidionini), and *Tetraopidion mucoriferum* (Thomson) (Neoibidionini). To our knowledge, all the species are endemic to South America, with *Eburodacrys assimilis* Gounelle and *Eburodacrys lenkoi* Napp and Martins only being known from Brazil [17]. The larvae of *Eburodacrys dubitata* White, *Eburodachrys flexuosa* Gounelle, *Compsibidion graphicum* (Thomson), and *Compsibidion sommeri* (Thomson) have been reported to infest several tree species in the family Fabaceae, including species in the genera *Acacia*, *Mimosa*, *Piptadenia,* and *Senegalia* [17]. However, to our knowledge, there is no published information on the biology or hosts of *E. assimilis*, *E. lenkoi,* and *T. mucoriferum*.

### 2.1. Source of Beetles for Collection of Headspace Volatiles

Adults of both sexes of *E. dubitata* were obtained from infested twigs of *Senegalia polyphylla* (DC.) Britton and Rose (Fabaceae) from a forest remnant of Cerrado (Brazilian savanna) located in Valentim Gentil, in the Brazilian state of São Paulo (20°22′19.2″S, 50°04′48.0″W), on 15 November 2015. The twigs were sawn into 30-cm pieces and housed in plastic containers under laboratory conditions (25 ± 1 °C, 60 ± 10% RH, 12 h photophase, and 5000 lux light intensity) in the Laboratory of Chemical Ecology and Insect Behavior, University of São Paulo, Piracicaba (~400 km from VG). Beetles emerged from twigs throughout January and February 2016. Adults of *E. assimilis* were collected with black light traps deployed near the above-mentioned forest throughout November 2016. Captured beetles were sent overnight by courier to Piracicaba.

For *E. flexuosa* and *E. lenkoi*, as soon as we found that the adults of both species were attracted by some treatment lures in the bioassays for *E. assimilis* (see Results), we deployed an extra set of four traps with the same attractants, to collect live adults of these species. We used cross-vane intercept panel traps (black corrugated plastic) hung from inverted L-shaped frames made from PVC pipe (for details see [14]). Rainwater was drained from the collection jars suspended below the traps by drilling 2-mm holes in the bottoms of the jars. We coated the surfaces of the traps and the internal surfaces of the collecting jars with an aqueous dispersion of Fluon^®^ (Insect-a-Slip; Bioquip, Rancho Dominguez, CA). Lures consisted of clear plastic press-seal sachets (polyethylene, 5 × 7.5 cm, 0.05 mm wall thickness; #01-816-1A, Fisher Scientific, Pittsburg, PA, USA), containing a cotton dental roll, loaded with a blend of 50 mg each of racemic 10-methyldodecanal and racemic 11-methyltridecanal, diluted in 900 μL of isopropanol. Lures were hung in the central open slot of traps, which were deployed in a forest remnant of Cerrado in Anhembi (~85 km from Piracicaba; 22°43′04.8″S, 48°10′26.4″W) and checked daily for captured beetles from 11 to 18 December 2018. Beetles were sent to Piracicaba on the day that they were captured.

The first adult of *C. sommeri* used for aerations was a male caught with a black light trap in Anhembi in 18 December 2018. Another three males and four females were collected in a remnant of the Atlantic Rainforest on the campus of the University of São Paulo, Piracicaba (22°42′43.4″S, 47°37′39.9″W), between 11 October and 18 November 2019. In this case, we used two traps baited with a blend of two synthetic pheromone candidates that were identified from the first specimen, i.e., 10-methyldodecanal and 10-methyldodecanol. The latter compound had been used in field bioassays in Anhembi (see details below), where it did not result in the attraction of any adults of *C. sommeri*, but it did attract adults of the congener *C. graphicum*. Consequently, the traps deployed in Piracicaba also made it possible to capture live adults of this species. In addition, we had previously collected two males of *C. graphicum* on citrus bushes in that location on 15 October 2019, and these were also used for pheromone collection.

Adults of all *Eburodacrys* species were sexed based on antennal length; antennae of males are more than twice their body length, whereas antennae of females are slightly longer than the body length [18]. Sexing of *Compsibidion* species was based on the morphology of antennomer III, which is thickened in males [19]. Males and females of all species were kept separately in plastic cages, with glass vials filled with 10% sugar solution for nourishment, for 48 h before being used for collection of headspace volatiles.

Voucher specimens of all the cerambycid study species have been deposited in the collection of the museum in the Department of Entomology and Acarology (USP/ESALQ), Piracicaba, SP, Brazil.

### 2.2. Collection of Headspace Volatiles from Beetles

Adult beetles were aerated individually or in groups of two of the same sex in custom-made cylindrical glass chambers (25 cm long × 6 cm i.d.), containing two glass vials with sugar solution. Volatiles emitted by beetles were trapped on 150 mg of 80/100 mesh HayeSep^®^ Q adsorbent (Supelco, Bellefonte, PA, USA) in a glass pipette (8.5 cm long × 0.5 cm i.d.), with the adsorbent held in place with glass wool plugs. Collectors were connected to the outlets of the chambers, with a screw cap fitted with a Teflon ferrule. Charcoal-filtered air was pushed through the chambers at 150 mL/min. Headspace volatiles were collected continuously from beetles for 48 h under the laboratory conditions described above, and collections were made as many as four times from each beetle. Aerations from chambers containing only feeder vials were made in parallel as controls to monitor for system contaminants.

Volatiles were eluted from collectors with three 500-μL aliquots of methylene chloride, into silanized amber glass vials. Each extract was then concentrated to ~500 μL under a gentle flow of N_2_ and stored at −30 °C until analysis. Overall, aeration extracts were obtained in the following numbers (males/females) from each species: *E. assimilis* (6/2); *E. dubitata* (16/9); *E. flexuosa* (8/4); *E. lenkoi* (12/5); *C. graphicum* (8/4); and *C. sommeri* (11/3). Sex-specific volatile compounds were detected in ~50% of aeration samples from males, but were not detected in any extracts from females.

### 2.3. Analysis of Extracts of Headspace Volatiles

Extracts of volatiles were initially analyzed in Brazil by gas chromatography with flame ionization detection (GC-FID), or by gas chromatography-mass spectrometry (GC-MS), to confirm the presence of sex-specific compounds. One microliter of extract was injected into a GC-2010 gas chromatograph (Shimadzu Corp., Kyoto, Japan), fitted with an HP5-MS capillary column (30 m × 0.25 mm i.d. × 0.25 µm film; Agilent Technologies, Santa Clara, CA, USA). Injections were made in splitless mode (purge valve off for 1 min), with an injector temperature of 250 °C and helium carrier gas at a linear velocity of 25 cm/s. In the same fashion, injections were made in a Shimadzu QP2010 Ultra GCMS (Shimadzu Corp., Kyoto, Japan), fitted with a nonpolar column (30 m × 0.25 mm × 25 µm film; Rxi-1MS; Restek, Bellefonte, PA, USA). Ion source and quadrupole temperatures were set at 250 °C. Mass spectra were recorded in electron impact mode (70 eV) from *m/z* 35–280. The GC oven was programmed from 35 °C for 1 min, increased to 40 °C at 2 °C/min, hold 1 min, and increased to 250 °C at 10 °C/min, hold 10 min. Representative extracts that contained detectable amounts of sex-specific compounds were sent to the University of California, Riverside (UCR), for identification of the sex-specific compounds. At UCR, extracts were reanalyzed with an Agilent 7820A GC, coupled to a 5977E mass selective detector. The GC was equipped with an HP-5 column (same dimensions as above), and samples were injected in splitless mode. Helium carrier gas was used, with an oven temperature program of 40 °C/5 min, 10 °C/min to 280 °C (hold 10 min). The injector, ion source, and quadrupole temperatures were 250, 230, and 150 °C respectively. Mass spectra were obtained with electron impact ionization (70 eV), scanning a mass range from 40–400 amu. Retention indices were calculated by comparison with a blend of straight-chain alkane standards.

### 2.4. Synthetic Chemicals

**Figure 1:**
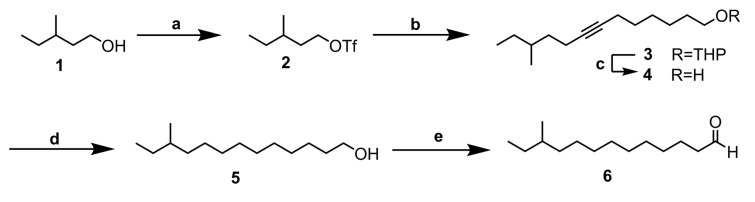
Synthesis of 11-methyltridecanal. (**a**) Tf_2_O, pyridine, CH_2_Cl_2_, −10 °C; (**b**) 2-(Oct-7-yn-1-yloxy)tetrahydro-2H-pyran, n-BuLi, THF; (**c**) PTSA, MeOH; (**d**) H_2_, 5% Pd/C, hexane; (**e**) NaOCl•5H_2_O, TEMPO, Bu_4_NHSO_4_, CH_2_Cl_2_.

10-Methyldodecanol and 10-methydodecanal were synthesized as described in [15]. The synthesis of 11-methyltridecanal was carried out as shown in Figure 1, and as described in detail in Figure S1.

### 2.5. Field Bioassays of Pheromone Candidates

We field-tested the synthetic version of the male-produced compound from *E. dubitata* (i.e., 11-methyltridecanal) in Valentim Gentil, from 5 December 2016 to 8 January 2017. We used cross-vane panel traps as described above, except that the collection jars were modified to hold 300 mL of an aqueous solution of dish soap and NaCl to kill and preserve the captured beetles. The lures (press-seal sachets with a cotton dental roll) were loaded with 50 mg of racemic 11-methyltridecanal, diluted in 950 µL of isopropanol. Control lures contained 1 mL of neat isopropanol. Traps were spaced ~15 m apart in four pairs (blocks), which were ~30 m apart. Previous studies have demonstrated that this is an ample distance between traps to prevent interference between treatments within a trap transect [20]. Pheromone and control treatments were assigned randomly to traps, and each pair contained one trap with each treatment.

Trap catches were counted every 2–3 d, at which time the preservative solution was replaced and treatment traps were switched in position within the block to control for positional bias. Lures were replaced every 15 d.

Field bioassays with *E. assimilis* were carried out in Valentim Gentil (from 28 November to 24 December 2018) and Anhembi (from 7 November 2018 to 5 January 2019). The lures contained 1 mL of a solution of synthetic pheromone candidates in isopropanol, with the following treatments: 1) racemic 10-methyldodecanal (50 mg); 2) racemic 11-methyltridecanal (50 mg); 3) natural blend of racemic 10-methyldodecanal (18 mg) and 11-methyltridecanal (50 mg), which mimics the ratio produced by adult males of *E. assimilis*; 4) 1:1 blend of these aldehydes (50 mg each); and 5) control (1 mL of isopropanol). The pheromone and control treatments were assigned randomly to traps within three (Valentim Gentil) and four (Anhembi) blocks, and each block contained one trap with each treatment. Traps were spaced ~15 m apart and blocks were ~30 m from each other. Traps were checked for beetles every 2–3 d in Valentim Gentil and every 15 d in Anhembi, following the procedures described above.

For the *Compsibidion* species, we had previously conducted two bioassays testing synthetic 10-methyldodecanol, before we had any evidence that *Compsibidion* species might produce this compound. This alcohol was originally identified from a North American cerambycid species (also tribe Neoibidionini; LMH and JGM unpublished data). Therefore, the bioassays in Brazil were intended to field screen this compound, in case any South American species might be attracted to it, as a lead to the identification of their pheromones. The compound was tested against controls in field bioassays in Anhembi from 24 November to 5 January 2019, using lures loaded with 50 mg of racemic 10-methyldodecanol, as described above. Control lures contained 1 mL of isopropanol. A total of two pairs of traps, each one containing one pheromone and one control treatment, were used. Traps were checked for beetles every ~15 d, as described above.

After the identification of male-specific volatiles from *C. sommeri* and *C. graphicum*, we followed up with bioassays testing 10-methyldodecanal and 10-methyldodecanol, individually and in blends. The treatment lures were: 1) a blend of 10-methyldodecanal (2.5 mg) and 10-methyldodecanol (50 mg), similar to the ratios produced by the beetles; 2) 10-methyldodecanol (50 mg); 3) 10-methyldodecanal (2.5 mg); and 4) control (1 mL of isopropanol). Treatments were assigned randomly to traps within six blocks, and each block contained representatives of all treatments. Traps were hung from tree branches at ~3 m from the ground and spaced 20 m apart, and blocks were 40 m from each other. Trap catches were tallied every 2–3 d from 12 November to 28 December 2019, following the same procedures used in the other bioassays.

### 2.6. Statistical Analysis

Differences between treatment means were tested separately for species represented by at least eight specimens per bioassay, using either the two-tailed Exact Binomial test (if there were only two treatments; [21]) or the nonparametric Friedman’s test (for greater numbers of treatments; PROC FREQ, option CMH; [22]), because field data violated assumptions of ANOVA [23]. Replicates were defined by spatial (block) and temporal (collection date) data. We included in each analysis only replicates that contained a minimum number of specimens, which ranged from 1 to 3 depending on species, in order to ensure a minimum number of replicates for a robust analysis (N ≥ 7 replicates). In recognition of the multiple statistical tests of treatment effects, significance levels were adjusted in the bioassays for *E. assimilis* in Valentim Gentil (α = 0.025; N = two analyses) and Anhembi (α = 0.017; N = three analyses), and in the bioassays for *Compsibidion* spp. (α = 0.017), according to the Bonferroni procedure [24]. Pairs of means were compared using the REGWQ multiple range test, which controls the Type I experiment-wise error rate [22]. The binomial test was performed with a spreadsheet available at http://www.biostathandbook.com/exactgof.html (accessed 31 January 2020).

The sex ratio of beetles captured by traps with the optimal attractant was compared to a nominal proportion of 0.5 with 95% Clopper–Pearson exact confidence intervals at 5% probability [25].

## 3. Results

### 3.1. Identification of Pheromone Candidates of Eburodacrys Species

**Figure 2:**
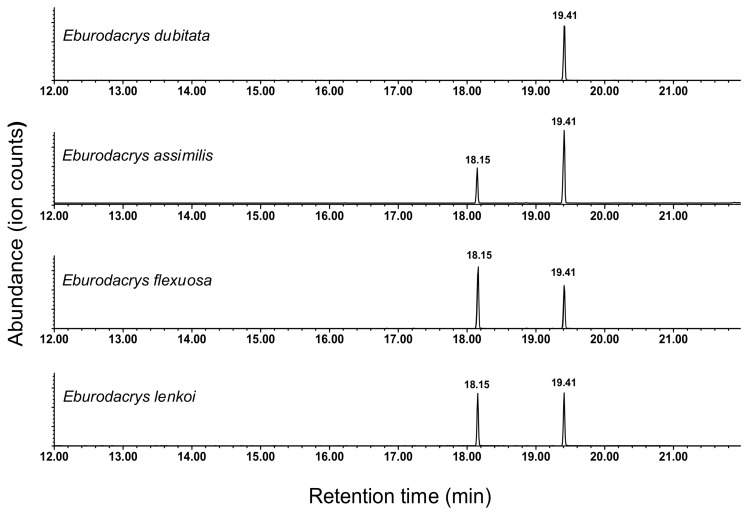
Representative total ion chromatograms of extracts of headspace odors from adult males of *Eburodacrys dubitata*, *Eburodacrys assimilis*, *Eburodacrys flexuosa*, and *Eburodacrys lenkoi*. The sex-specific peaks at 18.15 min (10-methyldocecanal) and 19.41 min (11-methytridecanal) were not present in equivalent extracts from females or system controls.

**Figure 3:**
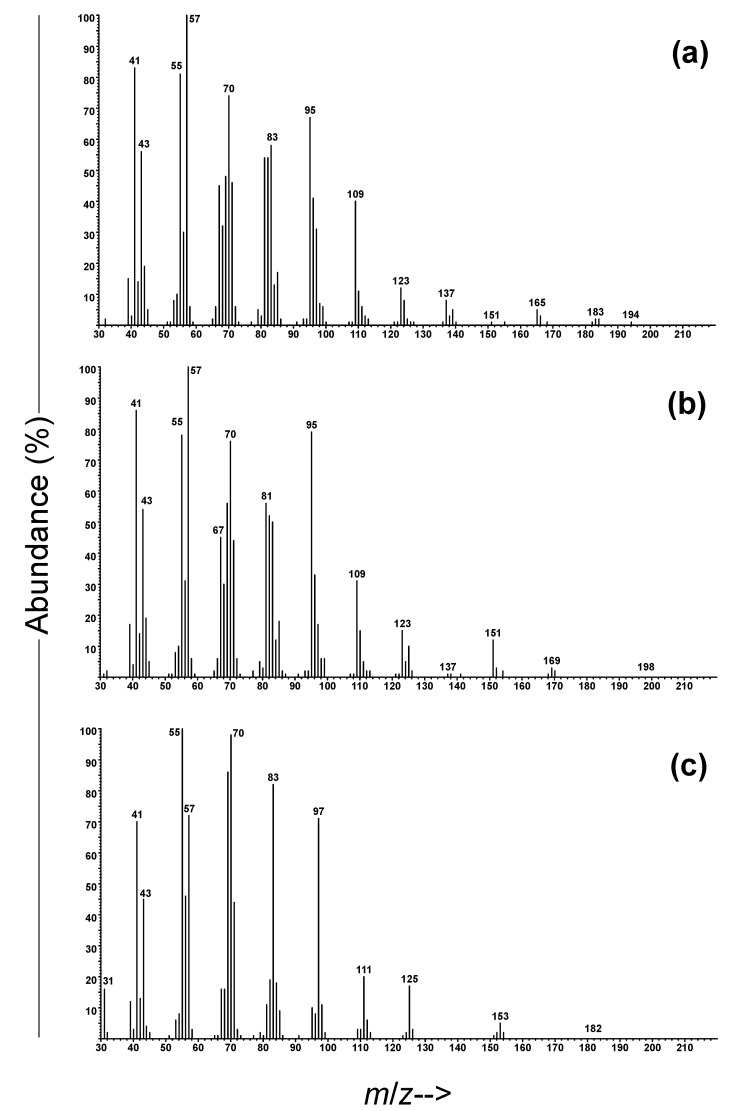
EI mass spectra of (**a**) 11-methyltridecanal, (**b**) 10-methyldodecanal, and (**c**) 10-methyldodecanol.

The analyses of extracts of headspace volatiles of adults of *E. dubitata* revealed the presence of a single prominent peak in extracts from males, which was absent from equivalent extracts from females and from system controls (Figure 2). The highest mass ion seen in the EI mass spectrum (Figure 3a) was *m/z* 194, with another small ion at *m/z* 183, with the 11 amu difference between them suggesting that the molecular ion was not visible, but might be *m/z* 212, with the loss of water (18 amu) giving the *m/z* 194 ion, and the loss of an ethyl group (29 amu) giving the *m/z* 183 ion, both common and plausible fragmentations. A possible molecular formula was thus suggested as C_14_H_28_O, with one site of unsaturation. The Kovats retention index of 1584 was substantially less than that of any monounsaturated straight-chain tetradecenols (range 1660-1695 KI units; [26]), but only 29 units less than tetradecanal (KI 1615), suggesting a possible branched chain aldehyde. Close examination of the mass spectrum showed a gap between *m/z* 137 and *m/z* 165 in the relatively regular clusters of ions separated by 14 mass units, suggesting the presence of a methyl branch that was lost along with the carbon to which it was attached, i.e., loss of 28 mass units, rather than 14. The 47 amu difference in mass between the *m/z* 165 ion and the putative molecular ion at *m/z* 212 could thus be accounted for by the sequential losses of water and an ethyl group from cleavage on one side of a methyl branch situated on carbon 3 from the end of the chain, whereas the *m/z* 137 ion could be accounted for by sequential losses of water and a *sec*-butyl radical from cleavage on the other side of the methyl group. Cumulatively, these data suggested that the structure was 11-methyltridecanal, and this was confirmed by synthesis of an authentic standard.

The other three *Eburodacrys* species produced blends of 11-methyltridecanal with a second compound, which was readily identified as the previously reported 10-methyldodecanal on the basis of matches of its mass spectrum (Figure 3b), and retention time with those of a synthetic standard. Thus, volatiles collected from adult male *E. assimilis* contained 11-methyltridecanal and 10-methyldodecanal in a ratio of 100:36.4 ± 0.3 (Mean ± SD, N = 5; Figure 2), whereas male *E. flexuosa* and *E. lenkoi* produced these two compounds in ratios of 100:61.5 ± 1.8 (N = 2) and 100:50.0 ± 4.2 (N = 3), respectively (Figure 2). In addition, extracts of *E. flexuosa* males contained a trace amount (0.5% of the 11-methyltridecanal peak) of a compound that was tentatively identified as the homolog 9-methylundecanal, on the basis of similarities between its mass spectrum and those of the two larger compounds, the presence of ions at *m/z* 109 and 137 with virtually no ions in between them, indicative of the methyl branch point, and its retention index of 1382, 100 units less than that of 10-methyldodecanal (KI = 1482).

### 3.2. Identification of Pheromone Candidates of Compsibidion Species

**Figure 4:**
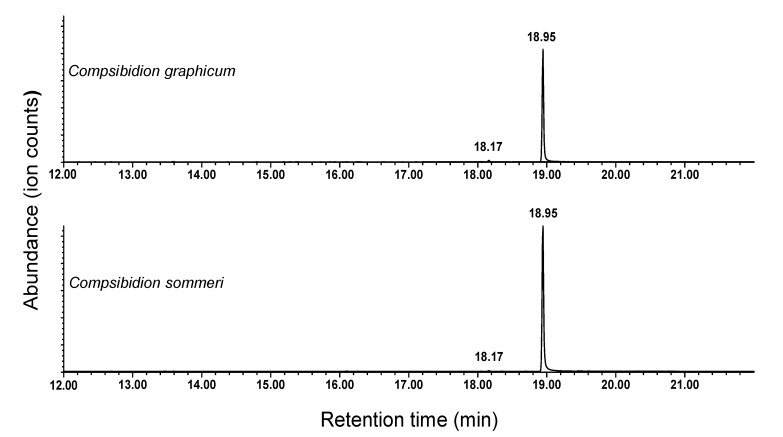
Representative total ion chromatograms of extracts of headspace odors from adult males of *Compsibidion graphicum* and *Compsibidion sommeri*. The male-specific peaks at 18.17 min (10-methyldodecanal) and 18.95 min (10-methyldodecanol) were not present in equivalent extracts from females or system controls.

Analyses of extracts of headspace volatiles from male *C. sommeri* by GC and GC-MS showed that adult males sex-specifically produced a blend composed of traces of 10-methyldodecanal and large amounts of a hitherto unknown component (ratio of 0.8 ± 0.4:100, N = 3; Figure 4). This compound was readily identified as the corresponding alcohol, 10-methyldodecanol. Specifically, the Kovats retention index was 65 units higher than that of 10-methyldodecanal, and the mass spectrum (Figure 3c) showed a number of ions that were two mass units larger than those in the mass spectrum of 10-methyldodecanal (e.g., 83 vs. 81, 97 vs. 95, 111 vs. 109). In particular, the diagnostic ions at *m/z* 125 and 153, with virtually nothing in between them, again signaled the position of the methyl branch on C10. This tentative identification was readily confirmed by matching the retention time and mass spectrum with that of a synthetic standard, which was available as an intermediate from the synthesis of 10-methyldodecanal. A blend of the same two compounds, in a similar ratio (0.9 ± 0.3:100, N = 4), was subsequently seen in extracts of volatiles from males of the congener *C. graphicum* (Figure 4).

### 3.3. Field Bioassays of Pheromone Candidates for Eburodacrys Species

In field tests conducted in Valentim Gentil in 2016, traps baited with synthetic racemic 11-methyltridecanal captured 76 adult males and females of *E. dubitata* (mean ± SE, 3.2 ± 0.3 beetles/replicate), versus no captures in control traps (means significantly different; exact binomial test: *p* < 0.0001). The sex ratio of captured beetles was significantly female biased (i.e., 63.1% females; 95% Clopper–Pearson exact confidence intervals: 0.51–0.74, *p* = 0.022).

**Figure 5:**
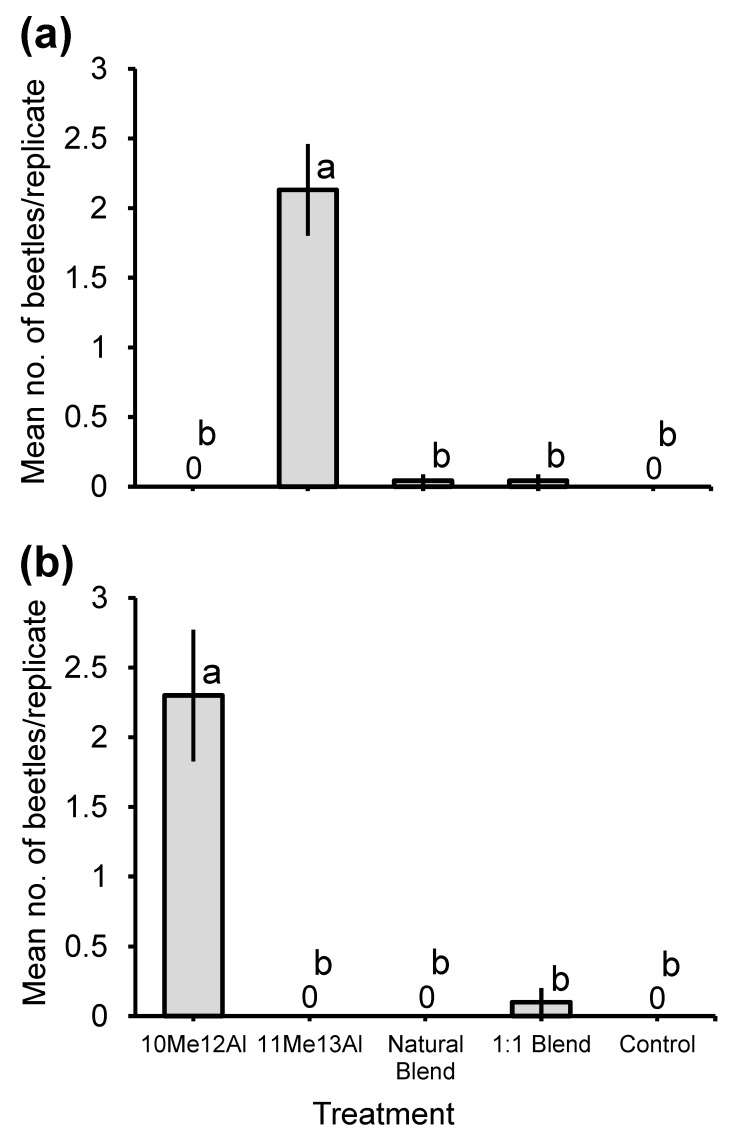
Mean (±1 SE) numbers of adult males and females of (**a**) *Eburodacrys dubitata* and (**b**) *Eburodacrys vittata* caught during bioassays testing the synthetic pheromone candidates as attractants for *Eburodacrys assimilis* in Valentim Gentil in 2018. Treatments: 10Me12Al = racemic 10-methyldodecanal; 11Me13Al = racemic 11-methyltridecanal; Natural blend = 36:100 blend of racemic 10-methyldodecanal and 11-methyltridecanal (ratio produced by adult males of *E. assimilis*); 1:1 Blend = 1:1 blend of the two aldehydes; and Control = neat isopropanol. Means related to the same species capped by different letters are significantly different (REGWQ multiple range test: *p* ≤ 0.05).

**Figure 6:**
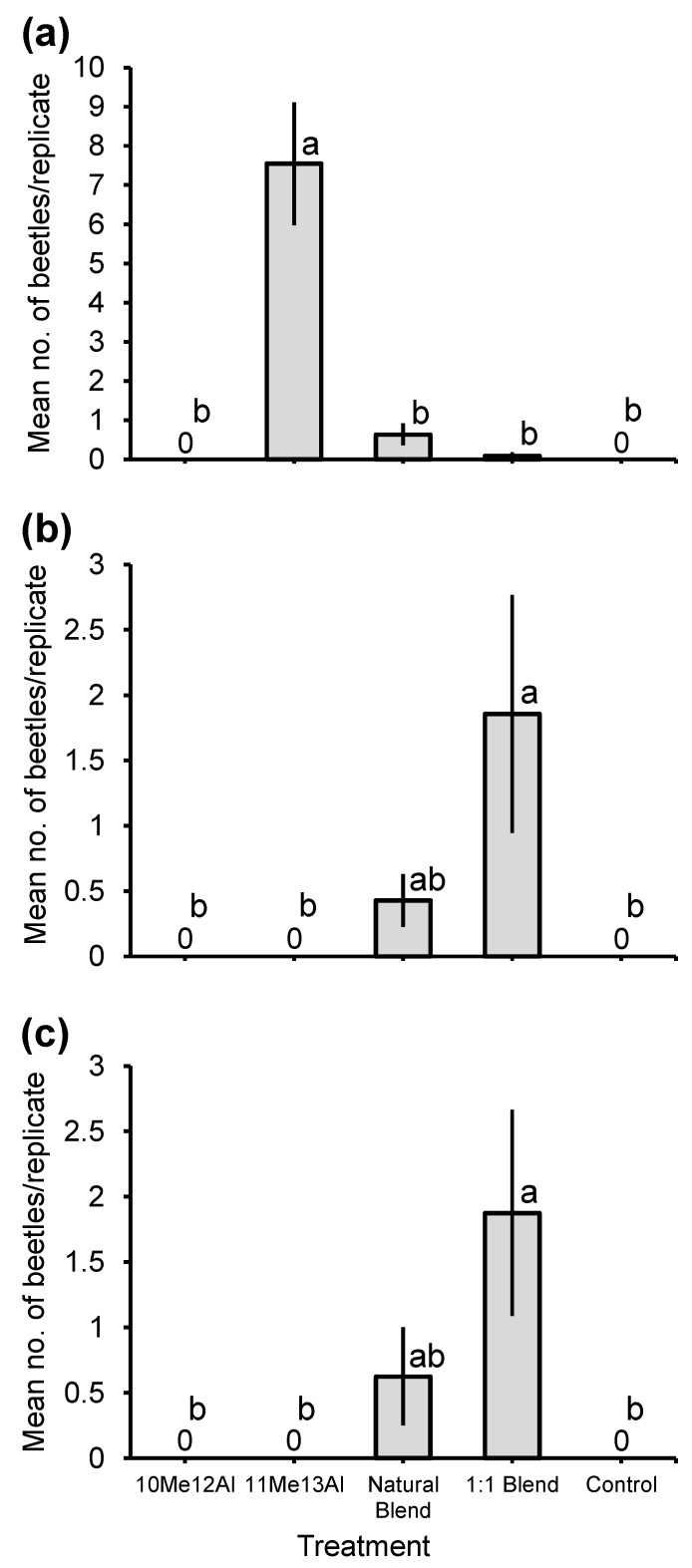
Mean (±1 SE) numbers of adult males and females of (**a**) *Eburodacrys dubitata*, (**b**) *Eburodacrys flexuosa* and (**c**) *Eburodacrys lenkoi* caught during bioassays testing the synthetic pheromone candidates for *Eburodacrys assimilis* in Anhembi in 2018. Treatments: 10Me12Al = racemic 10-methyldodecanal; 11Me13Al = racemic 11-methyltridecanal; Natural blend = 36:100 blend of racemic 10-methyldodecanal and 11-methyltridecanal (ratio produced by adult males of *E. assimilis*); 1:1 Blend = 1:1 blend of the two aldehydes; and Control = neat isopropanol. Means related to the same species capped by different letters are significantly different (REGWQ multiple range test: *p* ≤ 0.05).

Two field bioassays conducted in Valentim Gentil and Anhembi testing 10-methyldodecanal and 11-methyltridecanal, alone and in blends, resulted in capture of only one female *E. assimilis* at the first site. However, these bioassays yielded straightforward results for other congeners. Thus, the adults of *E. dubitata* were strongly attracted by 11-methyltridecanal in Valentim Gentil (Figure 5a; total of 51 beetles, 0 in controls) and in Anhembi (Figure 6a; total of 91 beetles, 0 in controls), compared to the other treatments (Q_4,115_ = 103.6, *p* < 0.0001; and Q_4,55_ = 36.3, *p* < 0.0001, respectively). The sex ratios of captured beetles were female-biased for both Valentim Gentil (69. 4% females; 95% Clopper–Pearson exact confidence intervals: 0.55–0.82, *p* = 0.0066) and Anhembi (66.3% females; Clopper–Pearson exact confidence intervals: 0.55–0.76, *p* = 0.0030). By contrast, traps baited with 10-methyldodecanal attracted significantly more adults of *E. vittata* in Valentim Gentil than the other treatments (Q_4,50_ = 44.3, *p* < 0.0001; Figure 5b; total of 24 beetles, 0 in controls). The sex ratio of captured beetles was ~0.5 (52.2% females; 95% Clopper–Pearson exact confidence intervals: 0.31–0.73, *p* = 0.83). Two female *E. vittata* were attracted to the same treatment in Anhembi (data insufficient for statistics).

Serendipitously, two additional *Eburodacrys* species were caught in significant numbers in Anhembi. That is, adults of *E. flexuosa* (total 16 beetles) and *E. lenkoi* (total 20 beetles) were strongly attracted to traps baited with a 1:1 blend of 10-methyldodecanal and 11-methyltridecanal, in contrast to zero captures in control traps, or traps baited with either compound alone (Q_4,35_ = 17.3, *p* = 0.0017; and Q_4,40_ = 20. 2, *p* = 0.0005, respectively; Figure 6b,c). Attraction to the 36:100 blend was intermediate, being not significantly different from either the controls or the 1:1 blend (Figure 6b,c). The sex ratio of beetles attracted by the 1:1 blend was significantly female biased in *E. flexuosa* (84.6% females; Clopper–Pearson exact confidence intervals: 0.55–0.98 *p* = 0.0126), whereas it did not differ from 0.5 in *E. lenkoi* (66.7% females; Clopper–Pearson exact confidence intervals: 0.38–0.88, *p* = 0.20).

### 3.4. Field Bioassays of Pheromone Candidates for Compsibidion Species

**Figure 7:**
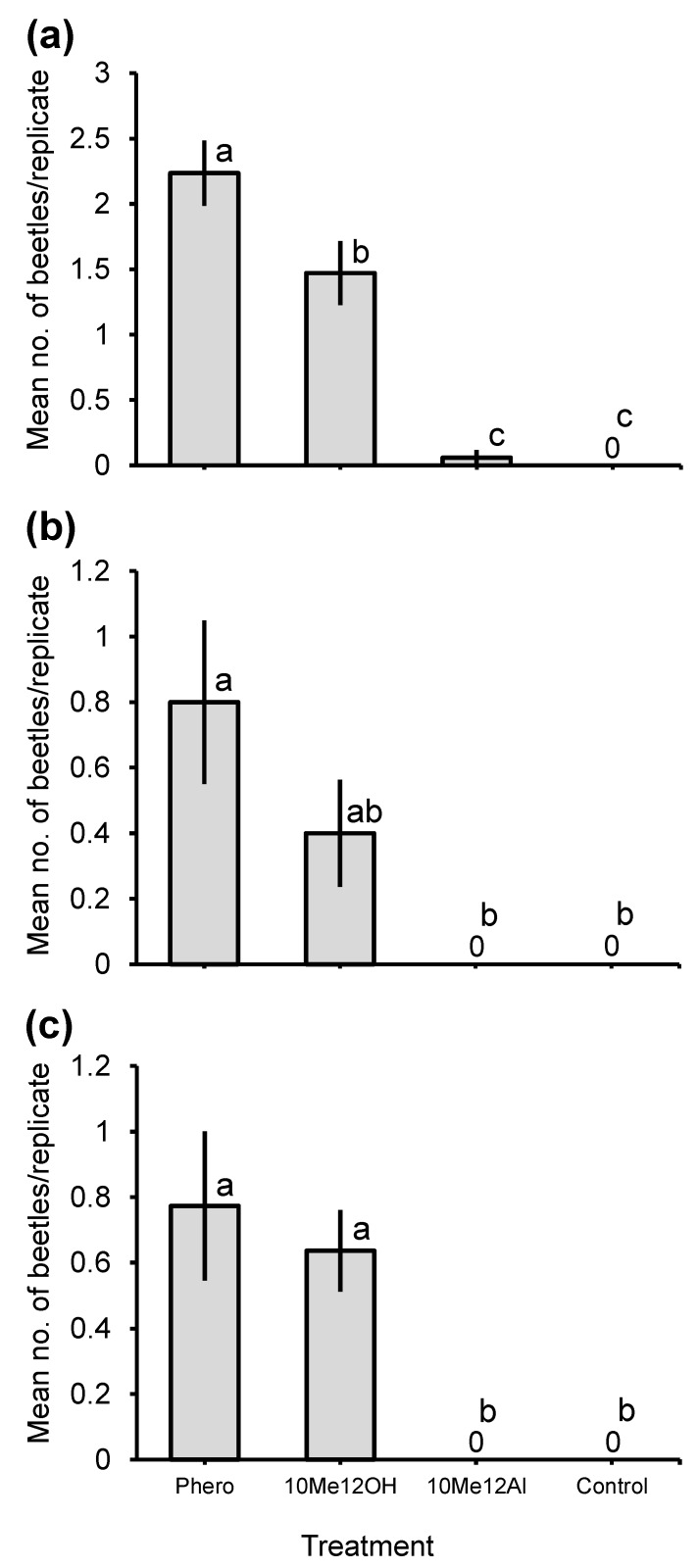
Mean (±1 SE) numbers of adult males and females of (**a**) *Compsibidion graphicum*, (**b**) *Compsibidion sommeri* and (**c**) *Tetraopidion mucoriferum* caught during bioassays in Piracicaba in 2019. Treatments: Phero = 5:100 blend of racemic 10-methyldodecanal and 10-methyldodecanol (approximate ratio produced by adult males of both *Compsibidion* species); 10Me12OH = racemic 10-methyldodecanol; 10Me12Al = racemic 10-methyldodecanal; and Control = neat isopropanol. Means related to the same species capped by different letters are significantly different (REGWQ multiple range test: *p* ≤ 0.05).

Previous screening trials conducted in Anhembi resulted in the capture of eight adult beetles (five males and three females) of *C. graphicum* in traps containing 10-methyldodecanol, which was significantly different from zero captures by controls (exact binomial test: *p* = 0.0078). In the bioassays conducted in Piracicaba, traps baited with a 20:1 ratio of 10-methyldodecanol plus 10-methyldodecanal caught significantly more adults of *C. graphicum* (total 108 beetles), than traps baited with the individual components or controls (Q_3,68_ = 50.9, *p* < 0.0001; Figure 7a). The sex ratio of captured beetles did not differ from 0.5 (54.7% females; Clopper–Pearson exact confidence intervals: 0.40–0.68, *p* = 0.49). Similarly, significantly more *C. sommeri* (total 12 beetles) were attracted to the 20:1 blend than to 10-methyldodecanal or the controls, but the blend was not significantly different than 10-methyldodecanol as a single component (Q_3,40_ = 14.3, *p* = 0.0025; Figure 7b). The sex ratio in the most attractive treatment was female biased (83.3% females; Clopper–Pearson exact confidence intervals: 0.52–0.98, *p* = 0.021). During these bioassays, another cerambycid species, *T. mucoriferum* (total 31 beetles), also in the tribe Neoibidionini, was attracted by both the blend (0.7 ± 0.2 beetles/replicate) and 10-methyldodecanol (0.6 ± 0.1 beetles/replicate; treatments not significantly different), in comparison to zero attraction to the other treatments (Q_3,88_ = 32.4, *p* < 0.0001, Figure 7c). The sex ratio in the most attractive treatment did not differ from 0.5 (35.3% females; Clopper–Pearson exact confidence intervals: 0.14–0.62, *p* = 0.23).

## 4. Discussion

Recently, 10-methyldodecanal has been been identified as an attractant pheromone for the South American cerambycid beetle *E. vittata*, and we initially thought that this structural motif might be species-specific [15]. However, the results presented here demonstrate that this is not the case, because this compound and the analogous alcohol, and the one carbon homolog 11-methyltridecanal, constitute aggregation-sex pheromone components of seven additional sympatric cerambycid species.

We first analyzed the pheromone chemistry of the congener *E. dubitata*, whose adult males sex-specifically produce 11-methyltridecanal as a single component, which attracted conspecific adults of both sexes in field bioassays. Concomitantly, we found that males of *E. assimilis* produce a blend of 10-methyldodecanal and 11-methyltridecanal. However, field trials with these compounds as single components and in blends failed to attract *E. assimilis*, but serendipitously, they offered new insights into the likely pheromone chemistry of two congeners, *E. flexuosa* and *E. lenkoi*. Both sexes of these species were strongly attracted to a 1:1 blend of 10-methyldodecanal and 11-methyltridecanal. We subsequently found that males of both species produced approximately equal amounts of these two homologous aldehydes. In addition, these bioassays confirmed that 10-methyldodecanal as a single component only attracts *E. vittata*, as previously found [15], whereas 11-methyltridecanal as a single component was specifically attractive to *E. dubitata*.

We also showed that males of *C. graphicum* and *C. sommeri* sex-specifically produce a blend of 10-methyldodecanol, with much smaller but crucial amounts of 10-methyldodecanal. In the field, traps baited with 10-methyldodecanol did attract adults of both species, but the attraction of *C. graphicum* increased significantly when small amounts of 10-methyldodecanal were included in the lure. During these field trials, adults of yet another species, *T. mucoriferum*, were significantly attracted to treatments containing 10-methyldodecanol, offering good circumstantial evidence that this compound might also be a pheromone component of this species as well.

Taken together, these results represent another demonstration that, analogous to other insect taxa, sympatric cerambycid species evolve specific pheromone channels from a basic pheromone motif, by varying the combinations and ratios of components, with either strong synergism between components, or conversely, strong antagonism by one or more components that limit the cross-attraction of species which share the main pheromone component. Further complexity in the signal can be created by using components of different chain lengths, and/or components with the same carbon skeleton, but different functional groups. In addition, another layer of signal complexity may be possible, because each of the four compounds described above can exist as (*R*)- or (*S*)-enantiomers. In fact, this may explain the lack of attraction of *E. assimilis* in field trials, despite the clear analytical evidence that it produces a blend of 10-methyldodecanal and 11-methyltridecanal. That is, in field trials to date, we have only tested racemic compounds, and if adults of *E. assimilis* are strongly inhibited by one of the enantiomers of either one of these compounds, this could explain the complete lack of attraction to lures formulated with racemic compounds.

To date, we have not been able to resolve the enantiomers of either the alcohols or the aldehydes by chromatographic methods, in part due to the large separation between the functional group and the methyl branch. Thus, it will likely be necessary to estimate which enantiomer of each compound is produced by each species through bioassays of the enantiomers, or possibly non-racemic mixtures of the two enantiomers of each component. Such tests may prove very revealing, given several recent examples demonstrating enantiomeric discrimination, and synergism between enantiomers, for several cerambycid species [14,27,28].

Our results also provide another example of the frequent strong conservation of pheromone motifs among congeners and even more distantly related cerambycid species in different tribes. The data reported here show that the 10-methyldodecyl and 11-methyltridecyl motifs are conserved within a number of South American species in the tribes Eburiini and Neoibidionini. However, we also have evidence that the 10-methyldodecyl skeleton may be used by some North American species in the tribe Neoibidionini as well (LMH unpublished data). Given the large numbers of species in these tribes [17], we fully expect that additional species will be found using these pheromone components. We plan to address the role of enantiomers in the pheromone blends of these and related species in ongoing studies.

The results described here also have important implications for insect surveillance efforts worldwide, whose goal is the detection of new incursions of invasive species. In particular, our results, along with previously published work [15], suggest that 10-methyldodecanal, 11-methyltridecanal, and their corresponding alcohols represent a pheromone motif that is used by cerambycine species in several different genera in at least two different tribes, on at least two different continents. Furthermore, because the chemistry of these pheromones is markedly different than the known pheromones of other cerambycid species, it seems highly likely that these compounds could be incorporated into generic lures containing pheromones of a number of species, with minimal chance of inhibiting attraction of other species. Thus, we anticipate that these new pheromones will be of substantial value and use to regulatory agencies charged with the detection and monitoring of invasive insect pests.

## 5. Conclusions

We have provided analytical and bioassay data, suggesting that pheromones based on 10-methyldodecyl and 11-methytridecyl structures may be widespread among at least two tribes within the subfamily Cerambycinae. The utilization of pheromone blends comprised of two components with different chain lengths and/or different functional groups, and in different ratios, represents an effective mechanism to limit deleterious cross-attraction between sympatric species which may share one or more compounds in their blends.

## Supplementary Materials

The following is available online at https://www.mdpi.com/2075-4450/11/4/222/s1, Figure S1: Synthesis of 11-methyltridecanal.
