# Supplementary material for: Variations on a Theme: Two Structural Motifs Create Species-Specific Pheromone Channels for Multiple Species of South American Cerambycid Beetles
Source: Insects. 2020 Apr 2;11(4):222. doi: 10.3390/insects11040222 (PMC7240461; doi:10.3390/insects11040222)
Supplement: Supplementary file 1 [file insects-11-00222-s001.pdf]

Synthesis of 11-methyltridecanal

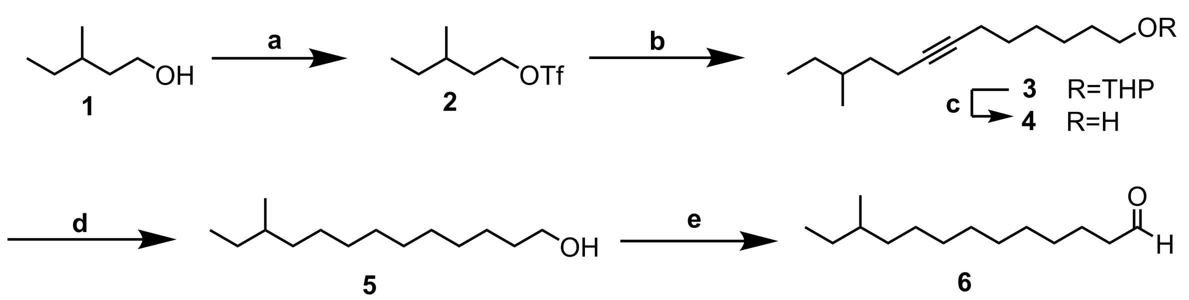

Figure S1. Synthesis of 11-methyltridecanal. **(a)** Tf_2O, pyridine, CH_2Cl_2, -10 °C; **(b)** 2-(Oct-7-yn-1-yloxy) tetrahydro-2H-pyran, n-BuLi, THF; **(c)** PTSA, MeOH; **(d)** H_2, 5% Pd/C, hexane; **(e)** $NaOCl•5H_2O$, TEMPO, Bu_4NHSO_4, CH_2Cl_2.

Commercially available starting materials and solvents were purchased from Sigma-Aldrich (Burlington, MA), Fisher Scientific (Pittsburg, PA), or GFS Chemicals (Powell, OH) and used without further purification. Tetrahydrofuran (THF) was distilled from sodium/benzophenone ketyl under an argon atmosphere. All reactions were performed under an argon atmosphere in oven-dried glassware. Solutions were dried over anhydrous Na_2SO_4 and concentrated by rotary evaporation under partial vacuum. Thin layer chromatography was performed on Polygram® SIL G/UV254 plates (Macherey-Nagel) with detection by immersion in a 10% phosphomolybdic acid solution in ethanol (Sigma-Aldrich), followed by heating. Vacuum flash chromatography was carried out with 230–400 mesh silica gel (Fisher Scientific). Mass spectra were obtained with a Hewlett–Packard (HP) 5890 GC (Avondale, PA) interfaced to an HP5970 mass selective detector, in EI mode (70 eV) with helium carrier gas. The GC was equipped with an HP5-MS column (25 m × 0.25 mm id × 0.33 µm film). ^1H and ^{13}C NMR spectra were recorded with a Bruker Avance Neo 400 (400 and 100.5 MHz, respectively) spectrometer (Bellarica, MA), as $CDCl_3$ solutions.

(±) 3-Methylpentyl triflate (2)

Pyridine (9.27, 115 mmol) and trifluoromethanesulfonic anhydride (23.20 mL, 138 mmol) were added sequentially to a cold (-10 °C) solution of (±) 3-methylpentanol (11.75 g, 115 mmol) in dry CH_2Cl_2 (300 mL). The reaction was stirred at -10 °C for 1.5 h and then diluted with pentane (600 mL) and stirred at rt for 30 min. The resulting slurry was filtered through a plug of silica gel (100 g) and the filter cake was washed with pentane/CH_2Cl_2 (2:1, 2 × 100 mL). The filtrate was concentrated to give 26.9 g (quantitative) of **2** as a colorless oil. This was used in the next step without characterization or further purification.

(±) 11-Methyltridec-7-yn-1-ol (4)

2-(Oct-7-yn-1-yloxy)tetrahydro-2*H*-pyran (20.1 g, 95.8 mmol) was dissolved in dry THF (300 mL) and cooled to -78 °C. A solution of *n*-BuLi in hexanes (2.57 M, 41.0 mL, 105 mmol) was added dropwise, and the resulting mixture was stirred at -78 °C for 30 min, then warmed to -10 °C for another 30 min. The mixture was again cooled to -78 °C, and a solution of **2** (26.9 g, 115 mmol) in dry THF (40 mL) was added dropwise over 30 min. The resulting mixture was warmed to -10 °C and stirred for 5 h. The reaction mixture was then quenched with saturated aqueous NH_4Cl and extracted with hexane (2 × 200 mL). The organic extracts were combined, washed with aq. $NaHCO_3$ (2 × 250 mL) and brine (2 × 250 mL), dried,

and concentrated. The residue was purified by vacuum flash chromatography (hexane/EtOAc 9:1) giving 25.6 g of **3** as a clear oil, contaminated with 8% of the THP protected 7-octyn-1-ol. The resulting product was taken up in MeOH (100 mL), *p*-toluenesulphonic acid (PTSA, 100 mg) was added, and the mixture was stirred at rt for 3 h until the deprotection was complete by TLC. Solid $NaHCO_3$ (1 g) was then added, the reaction was stirred 30 min, then concentrated, and the residue was diluted with water (200 mL) and extracted with hexanes (2 × 200 mL). The combined organic extracts were washed with brine (2 × 100 mL), dried, and concentrated. The crude product was purified by vacuum flash chromatography (hexane/ EtOAc 5:1) yielding 14.5 g of **4** contaminated with 7-octyn-1-ol. The concentrated material was purified further via Kugelrohr distillation. After removing a forerun, the product distilled at ~90 °C at 0.03 mm Hg, yielding 11.9 g (56.6 mmol, 59%) of pure **4** as a colorless oil. EI-MS (70 eV): 210 (M^+, 1), 195 (1), 181 (3), 163 (4), 153 (2), 135 (6), 122 (23), 107 (44), 95 (80), 81 (100), 67 (70), 55 (54), 41 (49). ^1H NMR (400 MHz, $CDCl_3$): δ 3.62 (t, 2H, *J*=6.7 Hz), 2.14-2.08 (m, 4H), 1.76 (m, 1H), 1.61 (m, 1H), 1.51-1.29 (m, 8H), 1.13 (m, 1H), 0.91 (d, 3H, *J*=6.8 Hz), 0.84 (t, 3H, 7.1 Hz). ^{13}C NMR (100 MHz, $CDCl_3$): δ 80.4, 79.8, 63.0, 35.7, 34.8, 32.6, 29.4, 29.2, 28.9, 25.4, 19.1, 18.9, 18.7, 11.5 ppm.

(±) 11-methyltridecan-1-ol (5)

5% Pd/C (600 mg) was added to a solution of alkynol **5** in hexanes (120 mL), and the mixture was stirred under H_2 for 6 h. The mixture was then filtered through a plug of celite, rinsing with hexane, and the filtrate was concentrated and the residue purified by Kuhgelrohr distillation to afford alcohol **5** (11.1 g, 91.5%, bp <100 °C at 0.05 mm Hg). EI-MS (70 eV): 213 (M^+-1, 1), 196 (1), 167 (8), 139 (11), 125 (10), 111 (38), 97 (72), 83 (81), 70 (100), 55 (81), 41 (51). ^1H NMR (400 MHz, $CDCl_3$): δ 3.62 (t, 2H, *J*=6.6 Hz), 1.67 (br s, OH), 1.53 (quint, 2H, *J* ~7 Hz), 1.38-1.14 (m, 17H), 1.04 (m, 2H), 0.91 (t, 3H, *J*=7.1 Hz), 0.83 (d, 3H, *J*=6.8 Hz). ^{13}C NMR (100 MHz, $CDCl_3$): δ 62.9, 36.8, 34.6, 32.8, 30.1, 29.74, 29.71, 29.66 (2C), 29.60, 27.3, 26.3, 19.4, 11.5.

(±) 11-methyltridecanal (6)

A 250 mL flask was charged sequentially with 45 mL CH_2Cl_2, sodium hypochlorite pentahydrate (2.71 g, 16.5 mmol), Bu_4NHSO_4 (0.25 g, 0.75 mmol), and TEMPO (46 mg, 0.3 mmol), and the mixture was cooled to 0 °C [20]. Alcohol **5** (3.21 g, 15 mmol) was then added over 20 min by syringe pump. After a further 10 min of stirring, the reaction was quenched by addition of saturated aqueous sodium thiosulphate, and the mixture was extracted with 100 mL hexane. The organic layer was washed with water and brine, dried, concentrated, and Kugelrohr distilled (bp ~85/0.05 mm Hg), yielding aldehyde 6 (2.6 g, 82%) as a clear oil. EI-MS (70 eV): 194 (1), 183 (2), 165 (6), 137 (9), 123 (13), 109 (41), 95 (69), 83 (59), 82 (54), 81 (56), 71 (46), 70 (75), 69 (48), 68 (33), 67 (45), 57 (100), 55 (81), 43 (55), 41 (83). ^1H NMR (400 MHz, $CDCl_3$): δ 9.75 (t, 1H, *J*=1.7 Hz), 2.41 (td, 2H, *J*=7.4, 1.7 Hz), 1.62 (m, 2H), 1.35-1.20 (m, 15H), 1.10 (m, 2H), 0.84 (t, 3H, *J*=7.1 Hz), 0.82 (d, 3H, *J*=7.2Hz). ^{13}C NMR (100 MHz, $CDCl_3$): δ 202.74, 43.81, 36.53, 34.29, 29.88, 29.53, 29.39 (2C), 29.35, 29.27, 26.99, 21.98, 19.10, 11.29 ppm.
